# Rhesus monkey model of liver disease reflecting clinical disease progression and hepatic gene expression analysis

**DOI:** 10.1038/srep15019

**Published:** 2015-10-07

**Authors:** Hong Wang, Tao Tan, Junfeng Wang, Yuyu Niu, Yaping Yan, Xiangyu Guo, Yu Kang, Yanchao Duan, Shaohui Chang, Jianpeng Liao, Chenyang Si, Weizhi Ji, Wei Si

**Affiliations:** 1 Yunnan Key Laboratory of Primate Biomedical Research, Institute of Primate Translational Medicine, Kunming University of Science and Technology, Kunming, Yunnan, Chin; 2 National Engineering Research Center of Biomedicine and Animal Science, Kunming, Yunnan, China; 3 Department of Hepatobiliary Surgery, The First People’s Hospital of Yunnan Province, Kunhua Hospital Affiliated to Kunming Medical College, Kunming, China

## Abstract

Alcoholic liver disease (ALD) is a significant public health issue with heavy medical and economic burdens. The aetiology of ALD is not yet completely understood. The development of drugs and therapies for ALD is hampered by a lack of suitable animal models that replicate both the histological and metabolic features of human ALD. Here, we characterize a rhesus monkey model of alcohol-induced liver steatosis and hepatic fibrosis that is compatible with the clinical progression of the biochemistry and pathology in humans with ALD. Microarray analysis of hepatic gene expression was conducted to identify potential molecular signatures of ALD progression. The up-regulation of expression of hepatic genes related to liver steatosis (CPT1A, FASN, LEPR, RXRA, IGFBP1, PPARGC1A and SLC2A4) was detected in our rhesus model, as was the down-regulation of such genes (CYP7A1, HMGCR, GCK and PNPLA3) and the up-regulation of expression of hepatic genes related to liver cancer (E2F1, OPCML, FZD7, IGFBP1 and LEF1). Our results demonstrate that this ALD model reflects the clinical disease progression and hepatic gene expression observed in humans. These findings will be useful for increasing the understanding of ALD pathogenesis and will benefit the development of new therapeutic procedures and pharmacological reagents for treating ALD.

Alcohol-induced chronic liver disease is a major cause of global morbidity and mortality^1^. In developed countries, up to 66% of all chronic liver disease is caused by alcohol consumption, and alcohol abuse accounts for nearly half of all deaths from liver disease^2^. Therefore, alcoholic liver disease (ALD) is a significant public health issue with heavy medical and economic burdens. Alcohol-induced chronic liver disease is a progressive disease with several histological stages: fatty liver (steatosis), alcoholic hepatitis, chronic hepatitis with hepatic fibrosis or cirrhosis, and hepatocellular carcinoma^3^. However, the aetiology of ALD is not completely understood. In contrast to other liver diseases, such as viral hepatitis, the medical treatment for ALD has not improved significantly in decades^2^. Currently, there are no effective, universally accepted therapies for ALD^4^,^5^. The development of ALD drugs and therapies is hampered by a lack of suitable animal models that replicate both the histological and metabolic features of human ALD^2^.

To investigate the mechanisms of ALD, significant progress has been made in developing animal models that show liver steatosis, hepatitis, fibrosis, cirrhosis and carcinoma, especially in rodents^6^,^7^. However, the argument has been made that mouse models cannot faithfully reflect human conditions and that non-predictive animal models may result in the clinical failure of new drugs or therapeutic approaches^8^,^9^. A systematic genomic study demonstrated that mouse models of inflammatory diseases poorly mimic human inflammatory disease conditions^10^. Considerable diversity in RNA expression exists between humans and mice, reflecting the fundamental physiological differences between these two organisms^9^. Therefore, “humans are not simply 70-kg mice”, either in terms of pharmacology or toxicology^8^, and suitable animal models that mimic clinical disease progression in humans are still needed. Because of their genetic, anatomical and physiological similarity to humans, nonhuman primates are essential and irreplaceable animal models in human disease research. Previous studies have shown that long-term alcohol intake induces fatty liver and hepatic fibrosis in baboons^11^,^12^. Chronic alcohol exposure also has been shown to lead to liver steatosis and fibrosis in rhesus monkeys that were fed with a 7% ethanol solution and a low essential fatty acid diet for five years^13^,^14^.

Gene expression profiling will define prominently expressed pathways and new molecular targets in ALD, and notable changes in the node gene expression in ALD patients’ liver biopsies have been detected^15^. Increasing our understanding of the pathogenesis of ALD will provide valuable insight for ALD prevention and treatment strategies. Therefore, to enable the systematic preclinical evaluation of the efficacy and safety of candidate drugs or therapies for human ALD, it is important that rhesus ALD models reflect both clinical disease progression and gene expression. However, the gene expression of chronic alcoholic liver injury in rhesus monkeys has not been studied. In the present study, we describe the development of rhesus models with liver steatosis, alcoholic hepatitis and hepatic fibrosis. We also detected the clinical progression of the biochemistry and pathology of ALD in these animal models. Moreover, to identify potential molecular signatures of disease progression and to compare gene expression patterns to those of humans with ALD, array-based mRNA analyses were conducted using animal liver tissue.

## Results

Sixteen rhesus monkeys were fed with an alcohol solution for 3 years. The mean daily alcohol intake over the 3-year period was 9.6 g/kg per day (range: 6.2–12.1 g/kg per day), or 24% of the total daily caloric intake (range: 16–42%). Three control animals were maintained on the same diet but without alcohol, and food intake was monitored daily.

### Ultrasound examination and histological features of alcohol-fed rhesus monkeys

**Figure 1:**
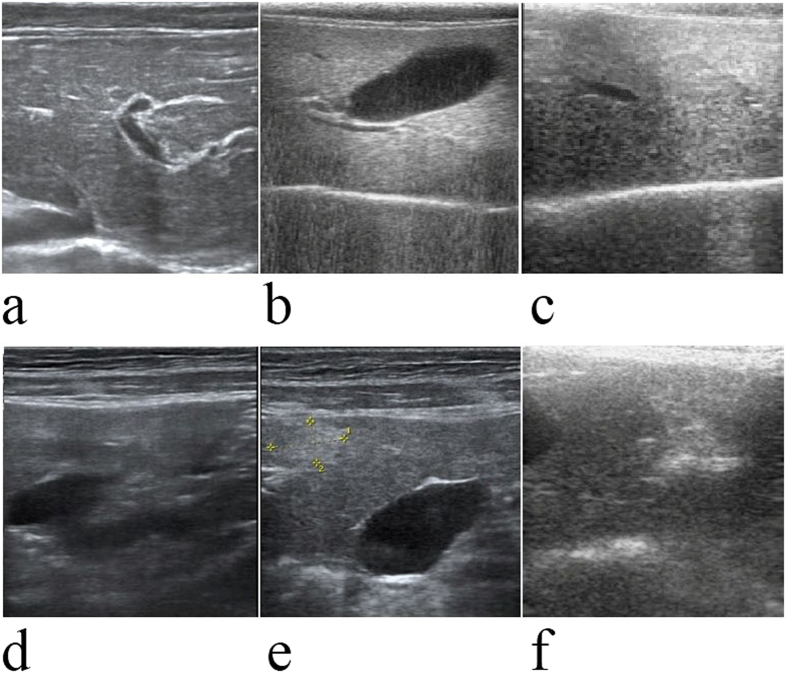
Ultrasound images of normal liver, fatty liver and liver with fibrosis. Normal liver tissue ultrasonogram (**a**): the liver capsule is neat and smooth with a linear, slim, strong echo, and there is a small gap in the linear echo at the peritoneal wall. The liver parenchyma spots are fine and uniformly distributed, the intrahepatic duct system is normal, the texture is clear, and entrant sound is good. Fatty liver tissue ultrasonogram (**b**,**c**): the liver capsule is neat and smooth, and the liver edge is obtuse. Intrahepatic echoes are fine and closely woven with uneven echoes. Intrahepatic vessels were significantly decreased. Liver fibrosis tissue ultrasonogram (**d**–**f**): the liver surface (**d**) is irregular and rough, and the liver parenchyma is uneven with irregular necrotic foci (**e**,**f**).

**Figure 2:**
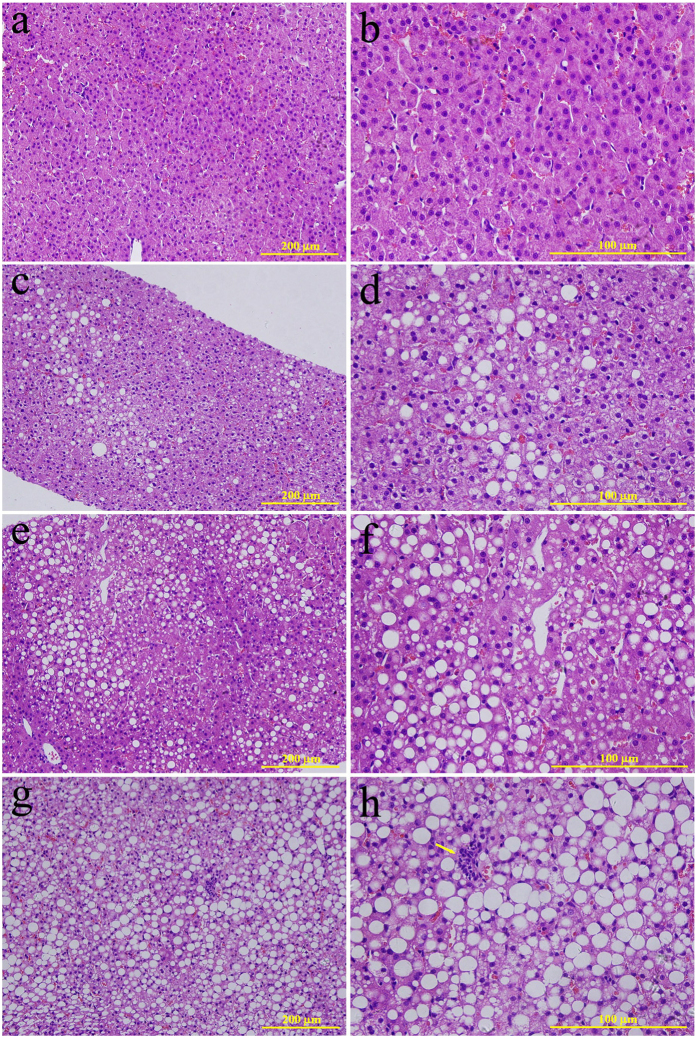
Development of liver steatosis. Histological features in normal rhesus monkey liver (**a**,**b**) and alcohol-fed rhesus monkeys with mild (**c**,**d**), moderate (**e**,**f**) and severe (**g**,**h**) liver steatosis (haematoxylin & eosin stain). Obvious hepatocellular vacuolization and macro- and micro-vesicular lipid accumulation can be observed in the liver hepatocytes. The arrow in Fig. 2h indicates infiltration of inflammatory cells.

**Figure 3:**
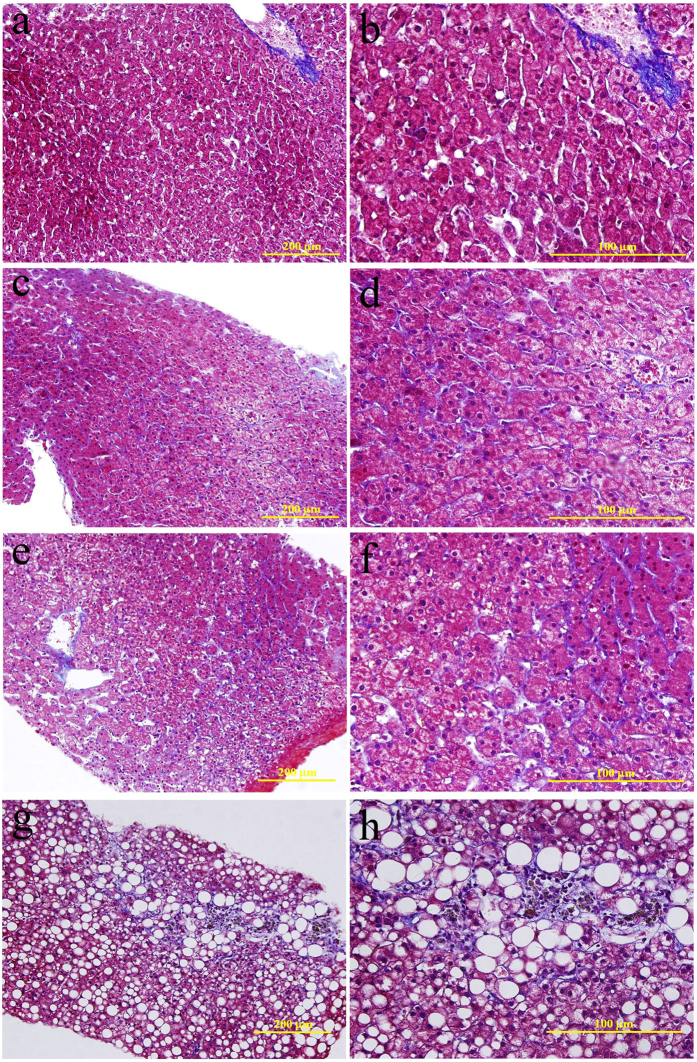
Histological features of normal rhesus monkey liver (**a,b**) and alcohol-fed rhesus monkeys with liver fibrosis (**c–h**). Deposited collagen fibres are densely stained by Masson’s trichrome.

Rhesus monkeys were maintained on an alcohol diet for 3 years. Alcohol-fed animals exhibited all of the stages of ALD, based on ultrasound examination and histological assessments. Liver ultrasound images of alcohol-fed rhesus monkeys presented clear liver steatosis and liver fibrosis, in contrast to non-alcohol-fed controls (Fig. 1). Histological examination of H&E stained liver sections revealed the development of mild, moderate and severe liver steatosis with necroinflammatory changes in alcohol-fed rhesus monkeys (Fig. 2). In contrast to the control animals, liver sections from alcohol-fed rhesus monkeys demonstrated steatosis, including hepatocellular vacuolization and macro- and micro-vesicular lipid accumulation in liver hepatocytes (Fig. 2c–h); inflammation and ballooning degeneration were also observed (Fig. 2h). Furthermore, histological examination of Masson stained liver sections demonstrated liver fibrosis in the alcohol-fed rhesus monkeys (Fig. 3). Individual monkey progressed through the stages of steatosis and steatohepatitis at different rates.

### Plasma chemistry features of alcohol-fed rhesus monkeys

**Figure 4:**
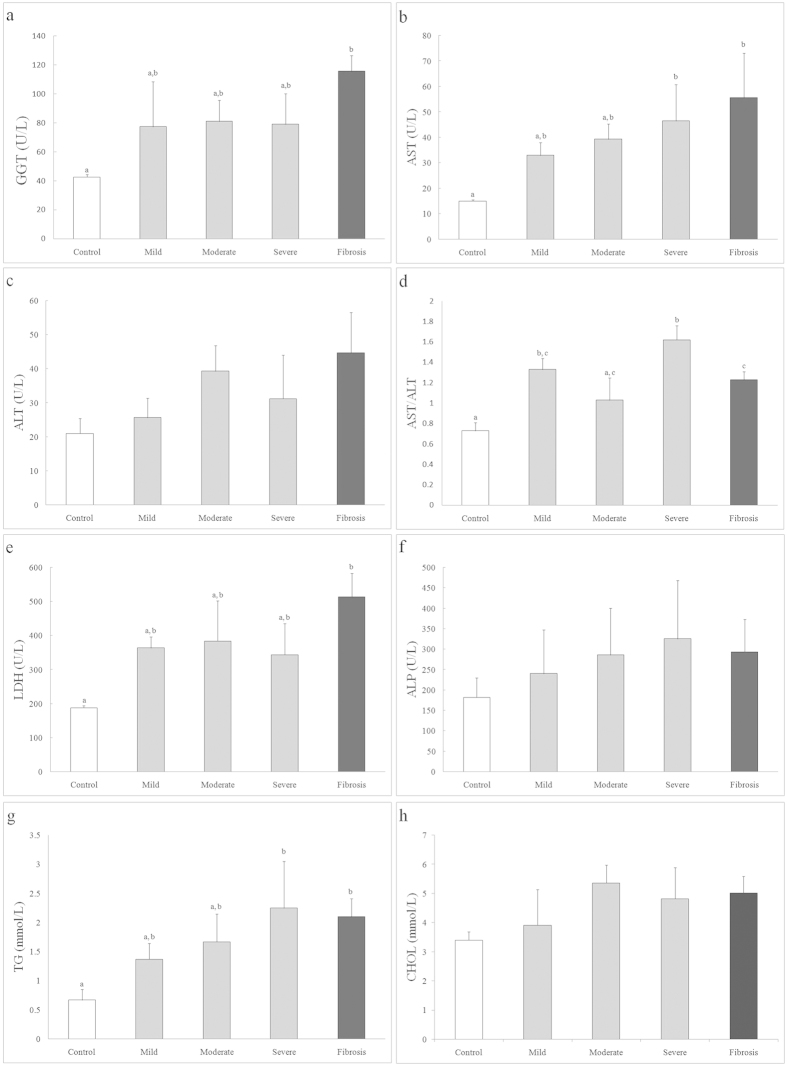
The serum plasma chemistry parameters for GGT, AST, ALT, AST/ALT, LDH, ALP, TG and CHOL(**a–h**) in rhesus monkeys with normal livers (open bar) and alcohol-fed rhesus monkeys with mild, moderate and severe liver steatosis (grey bars) and liver fibrosis (black bar). Different superscripts above columns indicate significant differences (P < 0.05).

**Figure 5:**
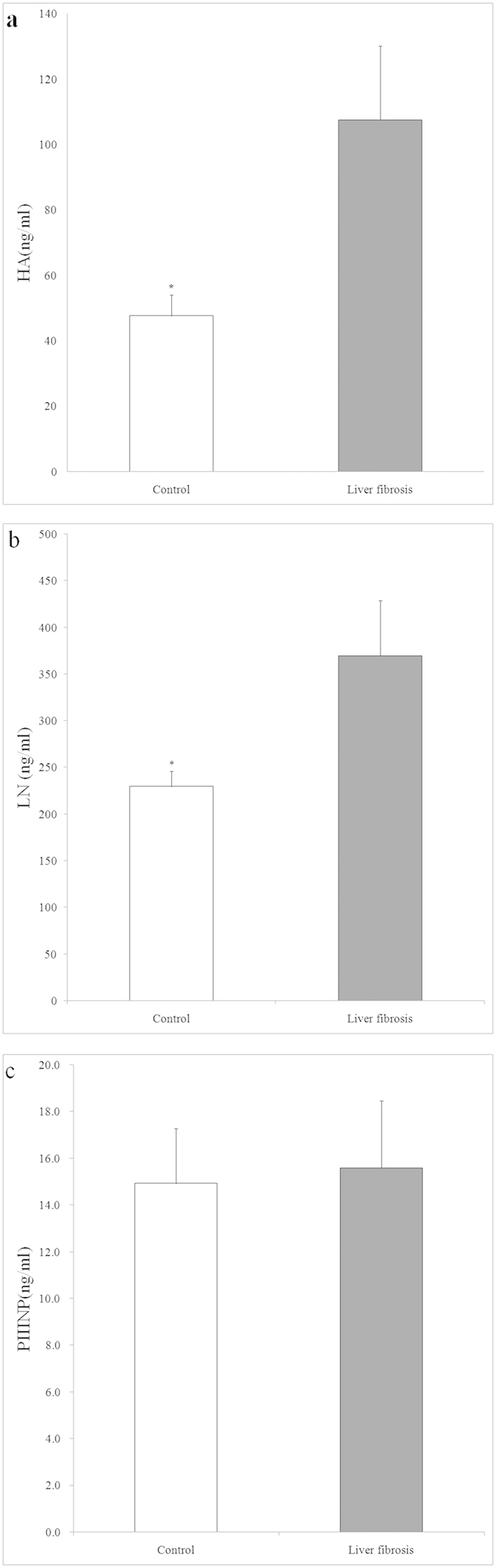
The liver fibrosis markers HA, LN and PIIINP(A to C) in rhesus monkeys with normal livers (open bar) and alcohol-fed rhesus monkeys with liver fibrosis (solid bar). *Significantly different between control and alcohol-fed rhesus monkeys with liver fibrosis (P < 0.05).

Based on the assessment of histological features in the liver sections, the alcohol-fed animals were categorized by disease stage according to severity: mild, moderate and severe liver steatosis and liver fibrosis. Liver function and blood serum lipids were also analysed, and the numbers of control monkeys, monkeys with mild, moderate and severe liver steatosis and monkeys with fibrosis were 3, 3, 3, 6 and 3 monkeys, respectively (Fig. 4a–h). Blood serum AST concentrations of animals with liver steatosis and fibrosis were elevated compared to the control group (P < 0.05), and an independent predictor of AST/ALT ratios (>1) for mild, moderate and severe liver steatosis and liver fibrosis groups indicated the presence of ALD in those monkeys (Fig. 4d). Meanwhile, plasma GGT concentrations in monkeys with liver fibrosis were significantly increased (P < 0.05), which was identified as an independent predictor of alcohol liver disease (Fig. 4a). The elevated level of plasma AST and the LDH of severe liver steatosis and liver fibrosis groups indicated liver damage in these animals (P < 0.05) (Fig. 4b,e). In addition, serum TG in alcohol-fed animals with symptoms of severe liver steatosis and liver fibrosis were also elevated compared to the control group (P < 0.05) (Fig. 4g). The serum indicators of liver fibrosis are shown in Fig. 5. The concentrations of serum HA and LN in animals with liver fibrosis that was confirmed by histological assessment were elevated compared to those of animals in the control group (P < 0.05). However, no significant difference in PIIINP was observed between control animals and animals with liver fibrosis.

### Hepatic gene expression changes in alcohol-fed rhesus monkeys with liver steatosis and liver fibrosis

**Figure 6:**
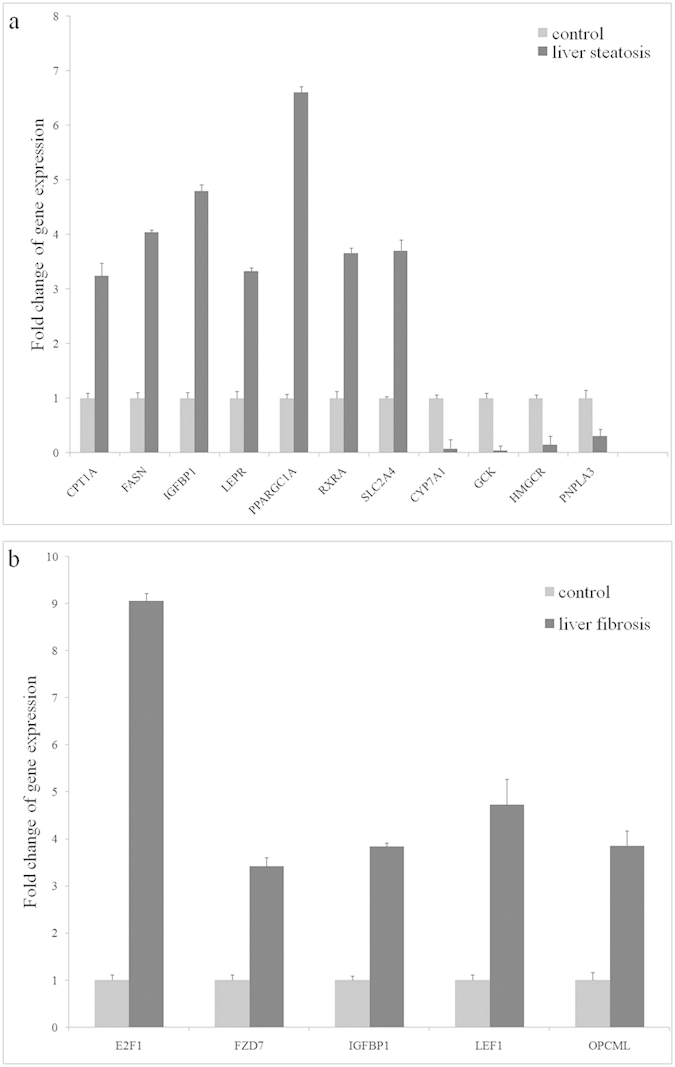
Microarray analyses of the change in hepatic gene expression between control rhesus monkeys and rhesus monkeys with liver steatosis (**a**) and liver fibrosis (**b**).

Liver tissues from 3 control monkeys and 6 monkeys showing severe liver steatosis were used for the analysis of hepatic gene expression using the Human Fatty Liver PCR Array; experiments were conducted in triplicate. Genes encoding proteins known to be related to beta-oxidation (CPT1A), lipid metabolism and transport (FASN) in metabolic pathways, adipokine signalling pathway (LEPR, RXRA), insulin signalling pathway metabolic pathways (IGFBP1, PPARGC1A) and a gene involved in non-insulin dependent diabetes mellitus (SLC2A4) were all elevated 3- to 6-fold. Several genes encoding proteins known to be related to cholesterol metabolism and transport (CYP7A1, HMGCR) and genes involved in non-insulin dependent diabetes mellitus (GCK, PNPLA3) were down-regulated 3- to 23-fold (Fig. 6a). Next, liver tissues from 3 control monkeys and 3 alcohol-fed monkeys showing liver fibrosis were used for the analysis of hepatic gene expression using the Human Liver Cancer PCR Array, and the experiments were conducted in triplicate. The expression of genes encoding proteins known to be associated with the cell cycle (E2F1), adhesion and proteolysis (OPCML) and hepatocellular carcinoma (FZD7, IGFBP1 and LEF1) was elevated 3- to 9-fold (Fig. 6b).

## Discussion

Alcoholic liver disease is a major cause of acute and chronic liver disease^16^. Animal models of ALD are important for understanding the mechanisms involved in disease progression and for developing diagnostic predictors or markers for therapeutic management. Progress has been achieved by using rodents and primates to study the pathology of ALD. Rodents remain the most accessible model for the study of ALD. However, alcohol-fed nonhuman primates that have developed liver disease may be a more representative model than murine models. In previous studies, it took up to 3–5 years to induce liver steatosis and fibrosis in rhesus monkeys by feeding them with a 7% ethanol solution at a consumption rate of 4.6 g//kg per day while on a low polyunsaturated fat or low n-3 fatty acid diet^13^,^14^. The amount of alcohol consumed and the duration of consumption are the most significant factors that affect the degree of hepatic dysfunction^16^. In the present study, we fed rhesus monkeys with an increased ethanol solution (25%) at an average consumption rate of 9.6 g/kg per day. This shortened the duration required to induce obvious liver steatosis and fibrosis to less than 3 years. Nonhuman primate models are very useful for increasing our understanding of ALD pathogenesis. The ALD rhesus model described in the present study can be used to facilitate preclinical assessments of safety and effectiveness and to benefit the development of new therapeutic procedures and pharmacological reagents designed for humans.

Histological evaluation of liver biopsies is a significant component of the diagnosis of steatohepatitis^17^. In our study, a total of 6, 3, and 3 of 16 rhesus monkeys showed severe, moderate and mild steatosis, respectively, which were accompanied by hepatocyte swelling and infiltrations of polymorphonuclear leukocytes, neutrophils and eosinophils. Two monkeys from the severe and moderate steatosis groups also showed liver fibrosis and a total of 3 monkeys showed liver fibrosis when assessed by histological evaluation. The other monkeys did not display obvious steatosis or liver fibrosis, but hepatocyte swelling and infiltrations, to different extents, were observed in all of the monkeys. Alcohol is primarily converted to acetaldehyde by alcohol dehydrogenase in the liver and acetaldehyde is subsequently converted to acetate by aldehyde dehydrogenase. In humans, it has been shown that alcohol dehydrogenase and aldehyde dehydrogenase exist as multiple isozymes that differ in their kinetic properties. The polymorphisms within the genes encoding these isozymes vary in their allele frequencies and may differentially influence the risk of developing alcohol diseases across ethnic groups^18^. Furthermore, polymorphisms in the genes encoding NF-κB subunits, interleukin (IL)-1β and IL-1 receptor antagonists, IL-s, IL-6, IL-10, CD14, toll-like receptor (TLR) 4, and PNPLA3 may also be associated with ALD and may modify ALD progression^2^. However, information regarding ALD-related genetic polymorphisms in rhesus remains undescribed. In our study, the individual difference in response to alcohol among these rhesus monkeys may be due to ALD-related genetic polymorphisms. In addition, the rhesus monkeys in the present study were fed daily with vegetables and fruits that contained propionic acid. Research has shown that undigested food is fermented in the colon by the microbiota, which gives rise to various microbial metabolites. Propionic acid is one of the principal metabolites produced and it can lower fatty acid content in the liver and plasma^19^. Therefore, it is possible that potential effects of ethanol on the pathology of steatosis and liver damage were counteracted by the specific diet of these monkeys in our study.

The pathophysiological significance of serum levels of biomarkers for the diagnosis of ALD remains unclear, and histological evaluation of liver biopsy continues to serve the main method for diagnosing ALD^2^,^17^. Serological tests can be used as clinical indicators for the diagnosis of human liver disorders. However, most serological predictors have fairly low sensitivities and specificities^20^. GGT and the AST/ALT ratio were identified as independent predictors of human ALD. In humans, when the AST/ALT ratio is >1.5, liver injury is considered to be ALD^21^,^22^. However, the use of serological tests as clinical indicators to diagnose ALD has not been studied in rhesus monkey models. In our study, alcohol-fed rhesus monkeys with severe steatosis and liver fibrosis showed increased GGT levels compared to the control groups. In our study, alcohol-fed rhesus monkeys were also found to have severe liver steatosis by histological evaluation, showing an average >1.5AST/ALT ratio. Studies in humans have indicated that the validity of the AST/ALT ratiois confounded by disease severity^23^. Our study on rhesus monkeys is consistent with the finding that a high AST/ALT ratio is frequently observed in human patients with advanced ALD^23^,^24^. In addition to increased GGT levels, rhesus monkeys found to have severe liver steatosis and liver fibrosis also showed increased levels of AST, LDH and TG. Furthermore, other serum markers, including HA, LN, IV-C, and PIIINP, which are markers of various aspects of collagen and extracellular matrix deposition, have been used to diagnose liver fibrosis as non-invasive surrogates for liver biopsy^25^. In our study, rhesus monkeys with liver fibrosis that was confirmed by histological evaluation showed significantly higher levels of serum HA and LA than did the control groups. However, the level of serum IV-C collected from rhesus monkeys with either liver fibrosis or a normal liver was below the level of detection of the chemiluminescence analyser. In humans, IV-C is not significantly correlated with the degree of liver fibrosis that results from long-term heavy alcohol consumption^26^, giving this marker limited value for predicting and diagnosing the stages of fibrosis compared to using a liver biopsy^26^,^27^. Therefore, the level of IV-C in control animals and animals with liver fibrosis was not compared.

Research on gene expression profiling of ALD will identity new pathways and differentially altered molecules. However, gene expression data from rhesus monkey liver disease models are sparse in the literature. The development of alcoholic fatty liver and fibrosis in rhesus monkeys has been reported^13^,^14^, but gene expression data from rhesus monkeys with chronic alcoholic liver injury has not been studied. The present study provides the first demonstration that alcohol induces changes in the hepatic gene expression of markers related to fatty liver and liver cancer in the livers of rhesus models. The fatty liver RT^2^ Profiler™ PCR Array used to determine hepatic gene expression in the present study has been successfully used in human hepatoma cells and rodent liver tissues^28^,^29^. In the present study, we identified seven up-regulated genes involved inoxidation, metabolic pathways, the adipokine signalling pathway, the insulin signalling pathway, and non-insulin dependent diabetes mellitus and four down-regulated genes involved in cholesterol metabolism and transport and non-insulin dependent diabetes mellitusin rhesus monkeys with liver steatosis. We also identified five genes that are associated with the cell cycle, adhesion, proteolysis and hepatocellular carcinoma that were up-regulated in rhesus monkeys with liver fibrosis. Accumulation of triacylglycerol in hepatocytes is one characteristic of liver steatosis. Differential expression of afatty acid oxidation marker (CPT1A) and of a fatty acid synthesis marker (FASN) have previously been reported in the livers of obese mouse and rat models fed with alcohol^30^,^31^. The expression of LEPR was found to be significantly altered in alcohol -fed mice and rats with liver steatosis^32^,^33^. RXRA was found to be up-regulated in hepatocellular carcinoma patients^34^. A high-fat diet greatly decreased the gene expression of markers associated with reverse cholesterol transport, including CYP7A1, in rat livers^35^, and a high sugar diet suppressed the expression of CYP7A1 in mouse livers^36^. HMGCR expression was correlated with cholesterol biosynthesis; a high cholesterol diet reduced the expression of HMGCR and CYP7A1 in the livers of Clock mutant mice^37^; and a high fat diet significantly decreased hepatic levels of HMGCR mRNA in rats^38^. PNPLA3 encodes a triacylglycerol lipase that mediates triacylglycerol hydrolysis. PNPLA3 is thought to be highly responsive to metabolic changes in hepatocytes in the liver, and relative changes in its expression level suggest an essential function in lipogenesis^39^. A genetic variant of PNPLA3 is associated with alcoholic and nonalcoholic liver disease^40^,^41^. Therefore, alterations to gene expression of markers associated with oxidation, lipid metabolism, cholesterol and adipokine signalling pathways in our rhesus models indicate an effect by alcohol on cumulative peroxisomal oxidation and lipogenesis in hepatocytes, which corresponds to observed increases intriacylglycerol and cholesterolserum levels. Glucokinase (GCK) regulates hepatic glucose metabolism and activates hepatic lipogenesis. Hepatic GCK activity was significantly reduced in mice fed with a highsucrose diet^42^. Abnormal expression of SLC2A4 was reported in patients who were diagnosed with obesity, insulin resistance and liver dysfunction^43^. PPARGC1A is the key gene that increases glucose metabolism and decreases lipid metabolism impairment in the liver^44^. Expression of PPARGC1A in the liver of mice was also found to be affected by alcohol consumption^45^. IGFBP1 has been identified as a biomarker that is associated with hepatotoxicity and alcoholic-induced liver injury^46^,^47^. The down-regulated expression of GCK and up-regulated expression of IGFBP1, PPARGC1A and SLC2A4 may indicate obesity and insulin resistance in rhesus models of liver steatosis. The expression levels of five genes that are associated with liver cancer were found up-regulated. The abnormal expression levels of these genes have been used as biomarkers for liver fibrosis and cirrhosis. E2F1 is a novel fibrogenic gene that regulates cholestatic liver fibrosis and could serve as a potential new diagnostic marker for nonalcoholic and alcoholic liver fibrosis and cirrhosis^48^. The expression level of FZD7 was up-regulated in hepatocellular carcinoma and has been considered to be an emerging target for cancer therapy^49^,^50^,^51^. IGFBP1 has been suggested as a diagnostic biomarker for hepatotoxicity and ALD^46^,^47^. The transcriptional activation of LEF1 facilitates tumour invasion and the occurrence of colorectal liver metastases is significantly correlated with the over expression of LEF1^52^,^53^. OPCML is a frequently methylated locusthat is associated with hepatocellular carcinoma^54^. The elevated expression of these genes possibly indicates the development of liver fibrosis and hepatocellular carcinoma in our rhesus models.

In conclusion, we characterized a rhesus monkey model of alcohol-induced liver steatosis, alcoholic hepatitis and hepatic fibrosis that is compatible with the clinical progression of biochemistry, pathology and hepatic gene expression in humans with ALD. This nonhuman primate model of liver disease will be useful for increasing the understanding of ALD pathogenesis and will benefit the development of new therapeutic procedures and pharmacological reagentsfor treating ALD.

## Methods

### Animals and alcohol administration

Nineteen adult rhesus monkeys (aged 9 to 12 years old) provided by Kunming Biomed International were used in this study. All of the animals were individually caged in an animal room with a 12 hour light: 12 hour darkness cycle. The temperature was maintained between 18 °C to 26 °C and with humidity from 40% to 70%. Animals were fed twice per day with commercial monkey chow (LabDiet, Harlan Laboratories, Inc., USA). Fresh fruits and vegetables were supplemented once per day. All procedures were approved by the Institutional Animal Care and Use Committee of Kunming Biomed International and were carried out in accordance with the Guide for the Care and Use of Laboratory Animals. The animal facility is accredited by the Association for the Assessment and Accreditation of Laboratory Animal Care International. Sixteen rhesus monkeys in the experimental group were given 24 h access to an ethanol solution that was sweetened with sugar. The beginning concentration of ethanol was 5% (v/v), and the ethanol concentration was increased by 5% each week until it reached a final concentration of 25%. The three control animals were given free access to water instead of alcohol.

### Blood collection and plasma chemistry tests

Blood samples were obtained from the femoral vein of rhesus monkeys that were anaesthetized by ketamine chloride (10 mg/kg; Shenyang Veterinary Pharmaceutical Inc., China) at approximately 9:00 am, after fasting for 14 h. For plasma chemistry tests, blood serum was separated by centrifugation at 3000 rpm for 15 min. The following plasma chemistry parameters were measured using a Roche Modular P800 automatic biochemical analyser (Roche Diagnostics Ltd., Basel, Switzerland): γ-glutamyl transpeptidase (GGT, U/l), lactate dehydrogenase (LDH, U/l), aspartate aminotransferase (AST, U/l), alanine aminotransferase (ALT, U/l), cholesterol (CHOL, mmol/l), triglyceride (TG, mmol/l). An ADC CLIA 400 chemiluminescence analyser (Addcare, Yantai, China) was used to measure serum plasma markers for liver fibrosis, which included serum hyaluronic acid (HA), Laminin (LN), collagen IV (IV-C), and procollagen III N-terminal peptide (PIIINP).

### Ultrasound examination and liver biopsy specimens

Ultrasonographic examinations were conducted every two months. After a minimum of 6 h of fasting, animals were anesthetized with ketamine chloride (10 mg/kg) via intramuscular injection (IM) and the lower abdominal and inguinal regions were shaved. The livers of all animals were imaged transcutaneously using a Diasus ultrasound system (Dynamic Imaging Ltd., Livingston, Scotland, UK), equipped with a 5 to 10 MHz linear-arraytransducer to grade the development of liver diseases. Before the biopsy procedure, rhesus monkeys were anesthetized by IM injection with ketamine chloride (10 mg/kg) and morphine hydrochloride (0.2 mg/kg, Northeast Pharmaceutical Group, China). Then, liver biopsy was performed using a Bard Monopty biopsy gun (Bard Biopsy Systems, Tempe, AZ., USA) loaded with a 16-gauge and echogenic-coated tip disposable biopsy needle (Bard Peripheral Vascular, Inc. USA) with a single pass by the percutaneous route in the right lower intercostal space, under the guidance of an ultrasound system. The biopsied liver tissue specimen was sectioned into two equal parts. One part was fixed in formaldehyde for subsequent histopathological analysis, and the other part was frozen in liquid nitrogen for subsequent RNA extraction.

### Histological analysis

Liver tissue samples collected by biopsies were carefully fixed in 10% neutral buffered formalin and embedded in paraffin. Paraffin-embedded tissues were sectioned and stained with haematoxylin and eosin (H&E) and Masson’s Trichrome (Masson) solutions. Specimens were graded with respect to fat accumulation and collagen deposition according to a previous study by an experienced veterinarian pathologist who was blinded to the treatment that the animals received^13^. Briefly, a mild (grade 1) fat score indicated that 10 to 15% of the hepatocytes contained vacuoles, a moderate (grade 2) fat score was assigned when vacuolization increased to 15 to 30%, and a severe (grade 3) fat score was assigned when the fat was distributed to more than 40% of the hepatocytes. Liver fibrosis was characterized by the appearance of densely staining collagen material.

### Microarray analyses of hepatic gene expression

Total RNA of liver tissues from various biopsy time points, according to the experimental design, were extracted by an RNeasy Micro Kit (Qiagen, Valencia, CA, USA). RNA quality was determined by spectrometry and agarose gel electrophoresis before PCR arrays. Reverse transcription was performed with ~1 μg RNA by using an RT^2^ First Strand Kit (SABioscience, MD, USA). Hepatic gene expression was determined with an RT^2^ Profiler™ Human Fatty Liver PCR Array and a Human Liver Cancer PCR Array (Qiagen, Frederick, MD, USA). A targeted array of 84 hepatic genes included genes involved in the regulation of adipokine and insulin signalling, metabolic enzymes and transporters and genes involved in inflammation and apoptosis was used to evaluate tissue from animals categorized by histopathological criteria to display characteristics of defined stages of liver steatosis. Another targeted array that profiled the expression of 84 genes involved in the progression of hepatocellular carcinoma and other forms of hepatocarcinogenesis was used to evaluate tissue from animals categorized by histopathological criteria to display liver fibrosis. The expression kinetics of 84 key genes that were implicated as potential biomarkers of fatty liver and liver cancer were simultaneously analysed from each PCR Array using the online software RT^2^ Profiler PCR Array Data Analysis Template v4.0 (Qiagen, Valencia, CA, USA).

### Statistical analysis

The plasma chemistry parameters were expressed as the means ± SEM. Plasma chemistry parameters (GGT, AST, ALT, AST/ALT ratio, CHOL, TG and LDH) in different groups were analysed by One-Way ANOVA (SPSS software, SPSS Inc., Chicago, IL, USA), and a least significant difference (LSD) test was used to identify differences. Student’s t test was used to detect differences in the levels of serum plasma markers between the control and experimental groups. For all analyses, P < 0.05 was considered to be significant.

## Additional Information

**How to cite this article**: Wang, H. *et al.* Rhesus monkey model of liver disease reflecting clinical disease progression and hepatic gene expression analysis. *Sci. Rep.* **5**, 15019; doi: 10.1038/srep15019 (2015).
